# Laparoscopic suturing learning curve in an open versus closed box trainer

**DOI:** 10.1007/s00464-015-4211-0

**Published:** 2015-05-05

**Authors:** S. P. Rodrigues, T. Horeman, M. S. H. Blomjous, E. Hiemstra, J. J. van den Dobbelsteen, F. W. Jansen

**Affiliations:** Department of Gynecology, K6-76, Leiden University Medical Center, PO Box 9600, 2300 RC Leiden, The Netherlands; Department of BioMechanical Engineering, Delft University of Technology, Delft, The Netherlands

**Keywords:** Training, Endoscopy, Surgical, Technical

## Abstract

**Background:**

The aim of this study was to examine the influence of training under direct vision prior to training with indirect vision on the learning curve of the laparoscopic suture task.

**Methods:**

Novices were randomized in two groups. Group 1 performed three suturing tasks in a transparent laparoscopic box trainer under direct vision followed by three suturing tasks in a standard non-transparent laparoscopic box trainer equipped with a 0° laparoscope. Group 2 performed six suturing tasks in a standard laparoscopic box trainer. Performance time, motion analysis parameters (economy of movements) and interaction force parameters (tissue handling) were measured. Participants completed a questionnaire assessing: self-perceived dexterity before and after the training, their experienced frustration and the difficulty of the training.

**Results:**

A total of 34 participants were included, one was excluded because of incomplete training. Group 1 used significantly less time to complete the total of six tasks (27 %). At the end of the training, there were no differences in motion or force parameters between the two groups. Group 2 rated their self-perceived dexterity after the training significantly lower than before the training and also reported significantly higher levels of frustration compared to group 1. Both groups rated the difficulty of the training similar.

**Conclusion:**

Novices benefit from starting their training of difficult basic laparoscopic skills, e.g., suturing, in a transparent box trainer without camera. It takes less time to complete the tasks, and they get less frustrated by the training with the same results on their economy of movements and tissue handling skills.

It is well established that the apprenticeship model is insufficient for acquiring minimally invasive surgical (MIS) skills and that basic MIS skills should preferably be trained in a non-clinical setting to ensure patient safety [1–4]. Simulator training programs are widely implemented into surgical, urological and gynecological resident curricula [5, 6]. Some of the challenges that MIS poses to surgeons and surgeons in training are:

- loss of depth perception and special orientation due to two-dimensional (2D) vision [7–9],
- perceived inversion of movement from the handle to the working end of the instrument “the fulcrum effect of the abdominal wall” [10–12],
- loss of haptic feedback due to resistance inside the trocars [13] and the use of long laparoscopic instruments [14],
- limited motion freedom and degrees of freedom (DOFs) due to the use of long rigid instruments [9, 12].

It is known that expert MIS surgeons learn to adjust to and compensate for these challenges. But when novices first start training of their basic MIS skills, they have to adjust to all these challenges at the same time making acquiring these skills a notoriously difficult task. Lack of training due to lack of time and motivation is a known problem, even though it is generally agreed among residents that simulation training of MIS skills is essential and should be obligatory [15–17].

Allowing novices to gradually adjust to the challenges MIS poses, by letting them train under direct vision before they switch to indirect vision, could be of benefit both in time consumption and in motivation. In theory, the main benefit would be that they do not have to compensate for the lack of depth perception and special orientation at the same time as learning how to manipulate the long rigid instruments and adjusting to the fulcrum effect. Compensating for a lack of depth perception requires the novice to use a variety of 2D cues such as light and shade, relative size of organs, organ interposition, texture gradient, aerial perception and motion parallax [7]. Because the effect is speculated to be more extensive in complex tasks compared to simple tasks, we chose to examine the effect of direct vision on the learning curve of the laparoscopic suture task. The aim of this study was to examine the influence of training under direct vision prior to training with indirect vision on the learning curve of the laparoscopic suture task. To this extend, we examine the consumed training time, the economy of movements, tissue handling skills and the novice’s opinion during the training.

## Methods

### Study population

Novices (i.e., first- and second-year medical students in the preclinical phase of their studies) were recruited by means of advertisement on bulletin boards in the Medical Faculty of Leiden and in the Medical Library of the Leiden University Medical Center (LUMC). They participated on a voluntary basis. Students with prior laparoscopic or simulator experience as well as students with prior experience in suturing more than once were excluded. In a previous study [18], statistical differences between force parameter outcomes were already detected with a sample size of six after training laparoscopic suturing with versus without visual feedback. Since the visual impact on the study groups in the current study was expected to be somewhat comparable, the aim was to recruit minimally six novices per group.

After enrollment, participants completed a questionnaire providing demographic information (i.e., gender, hand dominancy, prior suturing experience, experience in computer gaming and self-perceived dexterity on a seven-point Likert scale). Novices were randomly assigned to either the intervention or the control group using the Web site www.randomization.com. In both groups, novices performed one intracorporeal suture task six consecutive times (see detailed description below). The intervention group first performed the training task three times under direct vision in the interventional setup (trial one till three). Following these three interventional trials, the novices in the intervention group performed the training task three times in the control setup (trial four till six). The control group performed the training task six times in the control setup (trial one till six).

### Training setup

**Fig. 1:**
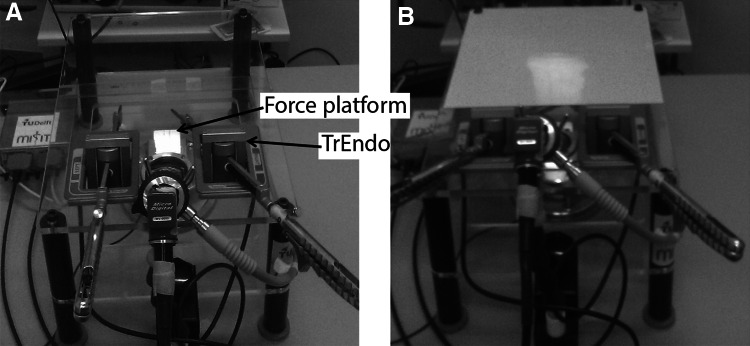
Physical box trainer (LUMC, Leiden). **A** Experimental (open) setup with transparent top so that the novices could look at the task under direct (3D) vision. **B** Standard (closed) setup: the transparent top was covered with a non-transparent plate and the image of a 0° scope was presented on a monitor in a fixed position (2D vision)

The laparoscopic box trainer was equipped with a TrEndo tracking system [19] for motion analysis and force measurement platform [20] for analysis of interaction forces (Fig. 1). Participants used two laparoscopic needle drivers (Ethicon, E705R), one in each hand. Since none of the subjects had previous experience with needle drivers, each subject had the opportunity to manipulate the buttons and handle for 5 min outside the training box before the first training task. Both the intervention group and the control group performed their training task in the same training setup with the mere difference that the intervention group could look through the transparent cover of the box during the intervention. By consequence, the effect of training with direct vision prior to training under regular conditions can be investigated.

### Task

**Fig. 2:**
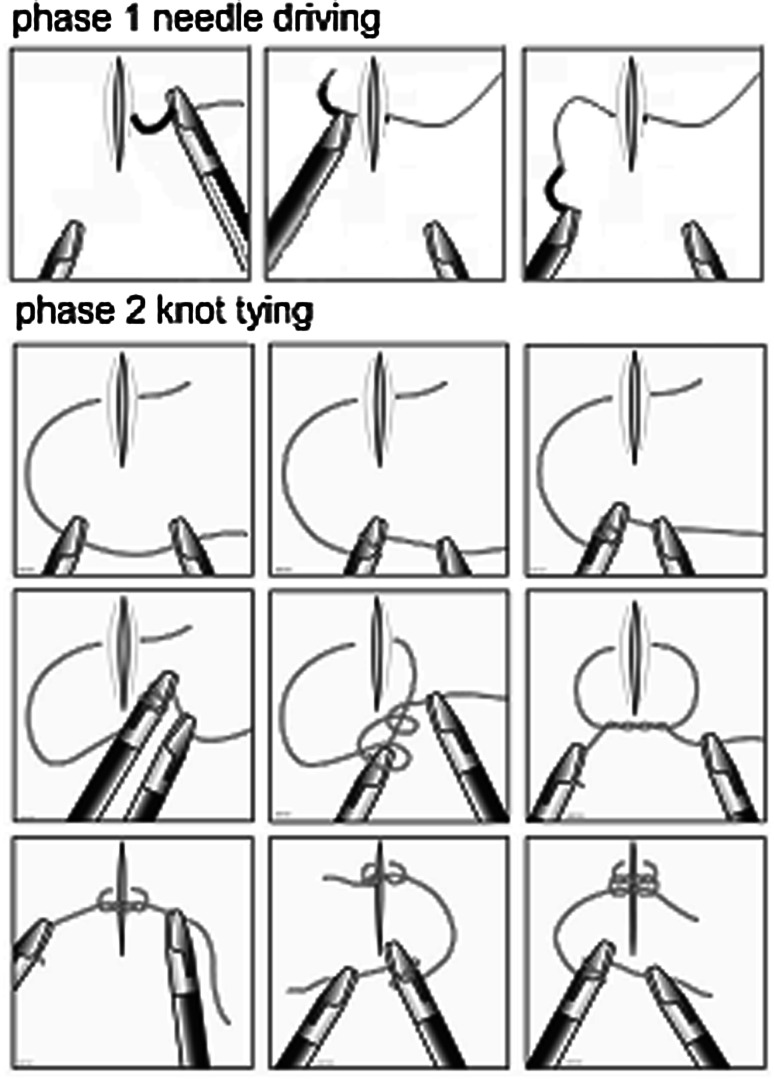
Step-by-step overview of the suture task performed in each trial. During phase 1, the needle is driven through the artificial tissue in a pre-marked area. During phase 2, a standardized three throw square knot is formed

Prior to training the task, a demonstration video was shown. Then, a step-by-step graphical explanation of the knot tying technique is shown in Fig. 2. Next, the video was demonstrated again while instructions were given about specific requirements (entry and exit point of the needle, when to use a double and single loop, etc.). The training task consisted of the placement of a simple suture followed by tying an intracorporeal knot in a standardized way. The exercise started with the needle (Vicryl 3-0 SH plus 26 mm, Ethicon, Johnson & Johnson) prepositioned in the needle driver and both needle drivers on a pre-marked place (start position). A proper bite (8 mm) has to be taken of the suturing pad in a pre-marked area. After pulling the needle and a substantial part of the thread through, a 3-throw knot was tied. This was done by making a double forward loop, followed by a single reverse loop and finally a single forward loop. During the training, the examiner coached a participant, if necessary, to perform the task correctly and to ensure that every suture was performed in the standardized manner. One examiner coached all participants throughout the entire trial.

For analysis of each trial (six per participant), the suturing task was divided into two phases:

1. *Needle driving phase* driving the needle through the artificial tissue.
2. *Knot tying phase* three throws to tie the knot.

We chose to divide the task into two phases because the force characteristics during the needle driving phase are essentially different from those during the knot tying phase. Namely, during the needle driving phase, continuous interaction with the artificial tissue causes different force parameters than during the knot tying phase, which does not include continuous tissue interaction.

Previous work by Hiemstra et al. [21] indicated that the steepest part of the suturing learning curve can be found within the first three trials. To ensure that most of the needed skills are acquired and the students stay motivated in our study, the training session was limited to six trials.

### Outcome measures

During the training session, the total time taken to perform each trial was recorded to get insight in the speed at which a trial could be successfully performed. Furthermore, the movements of the tip of the instruments were recorded with the TrEndo tracking device, developed at Delft University of Technology [19]. Consequently, motion analysis parameters were established of which we decided to analyze the following validated economy of movement parameters [21]:

1. *Path length* defined as the average length of the curve described by the tip of the right and the left instrument while performing the task (mm). Path length represents the economy of movements.
2. *Volume* defined as an ellipsoid around the standard deviation of the path length in three dimensions for both the right as well as the left instrument (mm^3^). Volume represents the precision of movements with less influence of single outliers.

A force measurement platform, developed at Delft University of Technology, was used to measure the maximum force and the mean absolute nonzero force in newton (N) as described by Horeman et al. [20]. Finally, we also chose to analyze the volume, which is calculated as the volume of an ellipsoid around the standard deviations in the three axes. This parameter was chosen because it represents the accuracy in which interaction forces are applied, i.e., tissue handling.

The suture quality was assessed immediately after completion of every task by the examiner on a three-point scale for:

1. correct entry and exit of the needle in the artificial tissue (2 points at exact entry and exit on the predetermined points, 1 point if entry and/or exit point deviated <1 mm, 0 points if entry and/or exit deviated more than 1 mm);
2. how tight the knot was pulled (2 points for correct tightness, 1 point if the knot was too tight with compression of the artificial tissue or too loose with a margin <1 mm, 0 points when the knot was too loose with a margin more than 1 mm).
3. slipping of the knot (2 points if for no slipping, 1 point for slipping <1 mm, 0 points for slipping more than 1 mm).

After the training, participants were asked again to complete a questionnaire in which they assessed self-perceived dexterity after the training, and they were asked to rate their experienced frustration and difficulty of the training all on a seven-point Likert scale.

### Statistical analysis

Statistical analysis of the data was done with the Statistical Package for Social Sciences (SPSS, version 16.0, Chicago, IL). Proficiency gain curves for time, path length, volume, maximum force, mean absolute nonzero force and force volume are plotted as means with 95 % CI. Differences in these parameters between the intervention and control group were determined with the independent samples *t* test (*P* < 0.05). Differences in suture quality between the two groups were determined with the Chi-square test (*P* < 0.05). Differences in assessed self-perceived dexterity, frustration and difficulty of the training were determined with the independent samples *t* test (*P* < 0.05).

## Results

### Participants

A total of 33 participants were included of which one was excluded because the training was not completed. The demographic information of the two groups is given in Table 1. There were no differences in gender, hand dominancy, prior suturing experience, experience in computer gaming and self-perceived dexterity prior to the training.

**Table 1:** Demographic information of the study population

	Group 1 Mean (*N* = 18)	Group 2 Mean (*N* = 14)	*P* value
Gender (*N*)			
Male	6	7	NS
Female	12	7	
Age (mean)	21.5	20.5	NS
Hand dominancy (*N*)			
Left	2	2	NS
Right	12	16	
Prior suturing experience (*N*)			
Yes	6	4	NS
No	8	14	
Experience in computer gaming (*N*)			
Yes	6	7	NS
No	8	11	

### Time

**Fig. 3:**
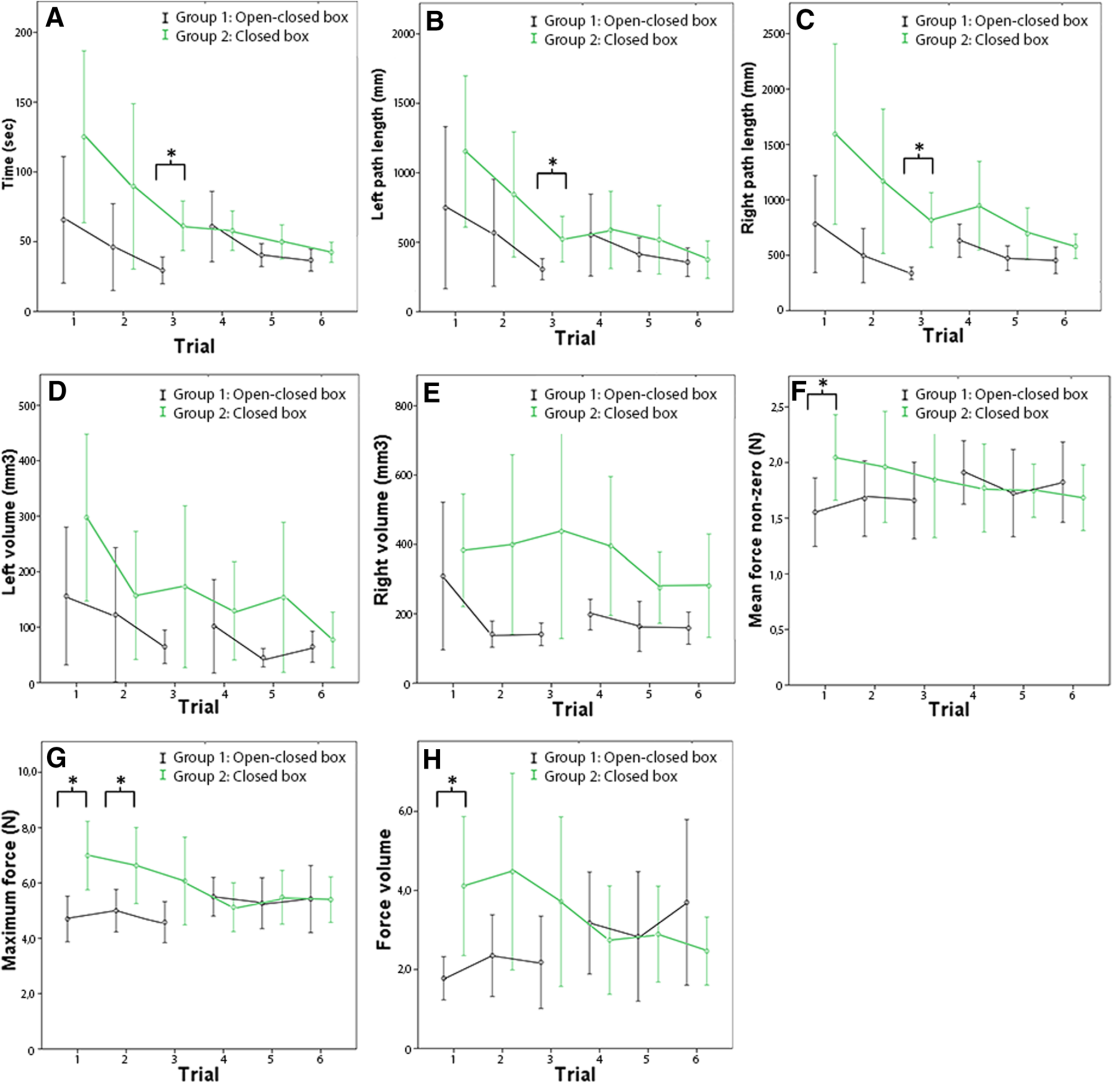
Learning curves of phase 1, the needle driving phase. In group 1, trial 3 is the last task in the open box trainer (with transparent top) before they switch to a standard (closed) box trainer. **A** Time. **B** and **C** Left and right path length. **D** and **E** *Left* and *right* volume. **F** Mean force nonzero. **G** Maximum force. **H** Force volume. *P < 0.05

**Fig. 4:**
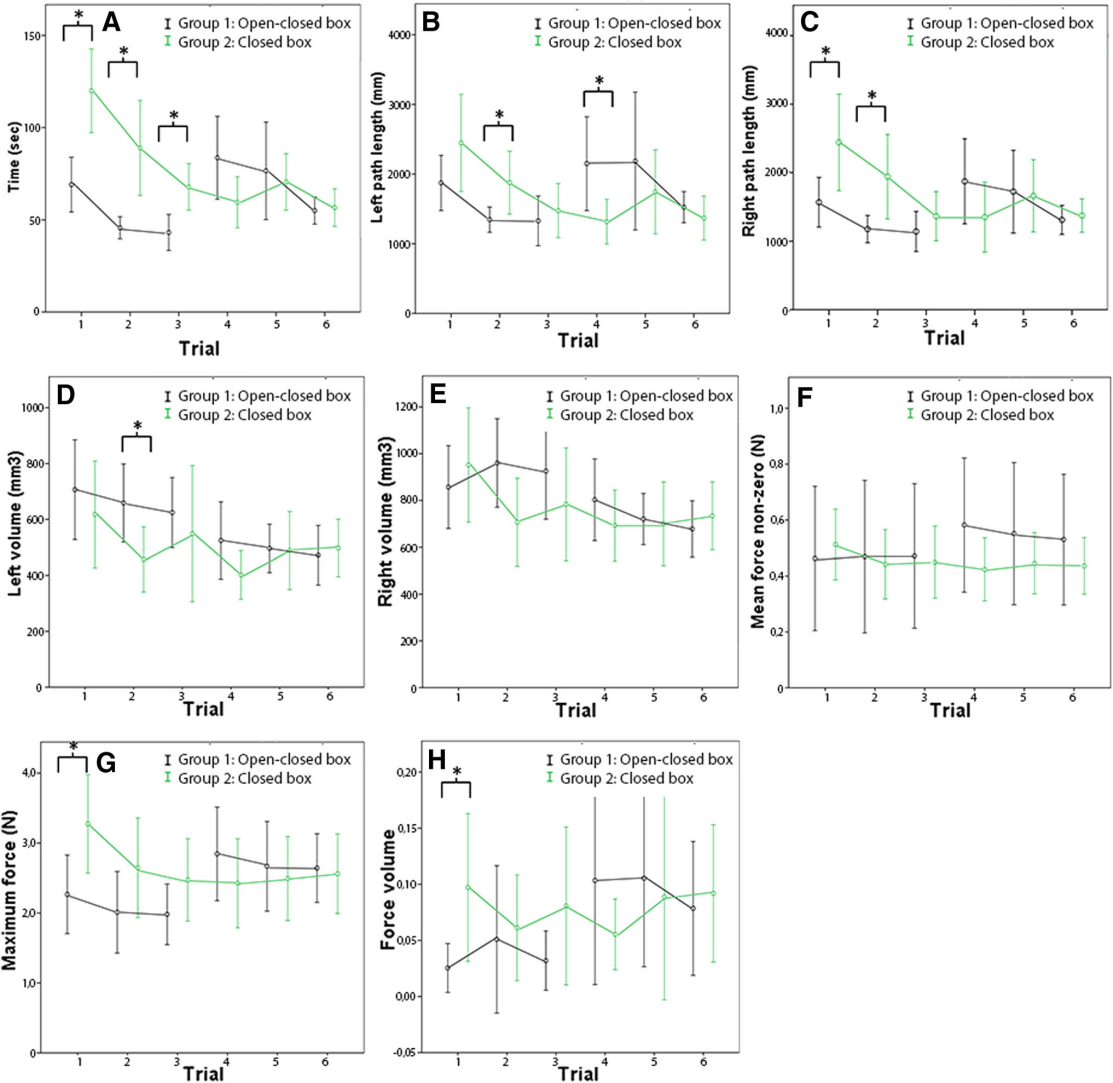
Learning curves of phase 2, the knot tying phase. In group 1, trial 3 is the last task in the open box trainer (with transparent top) before they switch to a standard (closed) box trainer. **A** Time. **B** and **C** *Left* and *right* path length. **D** and **E** *Left* and *right* volume. **F** Mean force nonzero. **G** Maximum force. **H** Force volume. *P < 0.05

The proficiency gain curves of time to complete the suturing task for both the needle driving phase and the knot tying phase are shown in Figs. 3A and 4A. The figures show that novices in the experimental setup group use significantly less time to complete the needle driving task during trial 3, e.g., the last task in the open box trainer before they switch to a standard box trainer. A significant amount of time is saved during the knot tying phase in the experimental setup. All knot tying tasks in the open box trainer are preformed significantly faster than in the standard box trainer (trail 1–3). In group 1, the total amount of time spend on performing all six trials ranged between 7 and 23 min with a mean of 11 min. In group 2, the total amount of time spend on performing all six trials was significantly higher and ranged between 10 and 26 min with a mean of 15 min. Group 1 uses 27 % less time than group 2 to perform the full training.

### Economy of movements

Figures 3B–E and 4B–E illustrate the proficiency gain curves of the path length and volume of the left and right hand. Differences between in motion parameters, if any, are seen in the first three trails when novices are training in different setups. These differences are washed out when group 1 switches from the experimental setup to the standard setup.

### Interaction forces

During the first trial, novices in the experimental setup use significantly less interaction forces than novices in the standard setup (Figs. 3G, H, 4G, H). This is seen in the needle driving phase as well as the knot tying phase. However, except for the maximum force used during trial, the needle driving phase of trial 2, there are no differences in interaction forces during trial two till six.

### Suture quality

There were no significant differences between the two groups in entry or exit points of the needle, tightness of the knot nor slipping of the knot as assessed by the examiner. There were no differences seen in any trial.

### Questionnaire

The average self-perceived dexterity of novices before training was similar in both groups. However, after training, the novices that had trained in the standard training setup rated their self-perceived dexterity significantly lower than novices who had trained on the experimental setup. Furthermore, novices that had trained in the standard training setup had experienced significantly higher levels of frustration than novices in who had trained on the experimental setup. Both groups rated the difficulty of the training similar. Mean given rates are given in Table 2.

**Table 2:** Results of the questionnaire on self-perceived dexterity, frustration and difficulty of the training

	Group 1 Mean (*N* = 18)	Group 2 Mean (*N* = 14)	*P* value
Self-perceived dexterity before training (mean)	5	4.67	NS
Self-perceived dexterity after training (mean)	4.79	3.94	0.023
Frustration assessed after training (mean)	2.43	4.06	0.003
Difficulty assessed after training (mean)	4.29	4.83	NS

## Discussion

The current study shows that novices that start training in an open box trainer perform better for the trials done in that box, but when they switch to the closed box their performance curves essentially overlap with those of students that train in closed box trainers alone. Novices that train in the open box spent 27 % less time training, while they achieve similar proficiency after six trials and subjectively report less frustration. This could affect novices’ motivation for training MIS skills. In standard laparoscopic trainings setups, novices have to adjust to all challenges MIS poses at once. By allowing them to start their training under direct vision instead of with 2D vision, they do not have to compensate for the lack of depth perception and special orientation at the same time as learning how to manipulate the long rigid instruments and adjusting to the fulcrum effect. As mentioned before, compensating for a lack of depth perception requires specific additional skills from the novice such as the using a variety of 2D cues such as light and shade, relative size of organs, organ interposition, texture gradient, aerial perception and motion parallax [7].

It is generally known that motivation for laparoscopic training is inversely related to laparoscopic experience. The motivation to learn can be intrinsic (from the trainee) and extrinsic [22]. The latter are exams, assessments, promotion, financial profits, prolonging registration, etc. These factors can be influenced by staff and program directors (e.g., by providing compulsory training time during working hours, inter-individual competitions and feedback). Several studies have focussed mainly on extrinsic motivational factors, for example, van Empel [15] has given trainees complete box trainers to make training at home available. Unfortunately, the average time trained at home (298.5 min; SD 383.1 min) was significantly lower than the self-reported desired training time (1687.6 min; SD 1225.9 min). Verdaasdonk [23] found that the competition element stimulated with a price was useful to attract mainly experienced trainees. These studies might suggest a significant role for intrinsic motivational factors. Intrinsic factors are motivators of such improvement of personal achievement (improvement of skills and knowledge), be prepared for new situations, security, but also fun and competition. These factors vary per person and are difficult to alter. The fact that novices in the group 2 (standard set up) rate their self-perceived dexterity after the training significantly lower than before the training together with the higher reported level of frustration in this group might suggest that the training in group 2 could decrease intrinsic motivation compared to the training in group 1 (training with direct vision first).

The importance of simulation training of MIS skills is stressed by residents themselves, and in surveys, they agree that simulation training is essential and should be obligatory [15–17]. However, voluntary simulation training remains a challenge. Besides a lack of motivation, a lack of time is the most common reported reason for not training [15–17]. This study shows that a considerate amount of time (27 %) can be spared by training under direct vision. A study by Hodgson [12], in which they examine the influence of various DOFs of laparoscopic instruments, has also shown that performing tasks under direct vision saves time (22 %). This time-saving effect was even larger (33 %) when in addition to direct vision extra DOFs were added.

When the optimal time is to switch from the experimental setup (direct vision) to the standard setup, indirect vision remains unclear. It can be speculated that most benefit will be gained when a trainee trains under direct vision until they master the skills to adjust to the fulcrum effect, the loss of haptic feedback and to the limited motion freedom and degrees of freedom (DOFs). Since trainees in this study switched to the standard setup after the third trial, we cannot answer this question.

A limitation of this study is the relatively small sample size. Therefore, we calculated the sample size needed to demonstrate a difference between the two groups for the different parameters (force as well as motion related) during the needle driving phase (phase 1) as well as the knot tying phase (phase 2) with a power of 80 % and a *P* value of 0.05 to demonstrate significance. The sample size needed varies between *N* = 261 (for phase 2; left path length) and *N* = 1.409.518 (for phase 1; right volume) The very large sample size needed for each parameter suggests that we would not find any differences, even if we would have extended our trial.

A limitation of the scoring system for suture quality is a lack of blindness of the scorer, which can result in bias. It was attempted to minimize bias due to lack of blinding by predefining the scoring system precisely. When it comes to the force and motion measurements, blinding is not an issue, as these measurements are objective and therefore not subjected to bias.

Several studies have shown advantages of 3D camera visualization over 2D visualization, mainly in saving time and surgeons’ preference, while others (using older visualization techniques) have reported equivalency in task performance [24]. This new technique is still relatively expensive, especially for training purposes. Providing physical box trainers with a transparent top is a cheap adjustment, and because it makes a camera and monitor system unnecessary, it should be possible to provide every starting trainee with their own training setup.

In conclusion, novices benefit from starting their training of difficult basic laparoscopic skills, such as suturing, in a transparent box trainer under direct vision. It takes them less time, and they get less frustrated by the training with the same end result on their economy of movements and tissue handling skills. Furthermore, it is a cheap adjustment to the standard box trainer setup, making it possible to provide trainees with their own training setup for the start of their basic laparoscopic skills training.
